# Enhanced bone regeneration via local low-dose delivery of PTH_1-34_ in a composite hydrogel

**DOI:** 10.3389/fbioe.2023.1209752

**Published:** 2023-07-03

**Authors:** Shanyong Zhang, Lei Ding, Gaoyang Chen, Jiayin Zhang, Wanbao Ge, Yuan Qu

**Affiliations:** ^1^ Department of Spine Surgery, The Second Hospital of Jilin University, Changchun, China; ^2^ Department of Rehabilitation, The Second Hospital of Jilin University, Changchun, China; ^3^ Shenzhen Key Laboratory of Musculoskeletal Tissue Reconstruction and Function Restoration, Department of Hand Surgery, Shenzhen People’s Hospital, The First Affiliated Hospital of Southern University of Science and Technology, Jinan University Second College of Medicine, Shenzhen, China

**Keywords:** PTH, bone regeneration, hydrogel, strontium, hydroxyapatite

## Abstract

Introducing bone regeneration–promoting factors into scaffold materials to improve the bone induction property is crucial in the fields of bone tissue engineering and regenerative medicine. This study aimed to develop a Sr-HA/PTH_1-34_-loaded composite hydrogel system with high biocompatibility. Teriparatide (PTH_1-34_) capable of promoting bone regeneration was selected as the bioactive factor. Strontium-substituted hydroxyapatite (Sr-HA) was introduced into the system to absorb PTH_1-34_ to promote the bioactivity and delay the release cycle. PTH_1-34_-loaded Sr-HA was then mixed with the precursor solution of the hydrogel to prepare the composite hydrogel as bone-repairing material with good biocompatibility and high mechanical strength. The experiments showed that Sr-HA absorbed PTH_1-34_ and achieved the slow and effective release of PTH_1-34_. *In vitro* biological experiments showed that the Sr-HA/PTH_1-34_-loaded hydrogel system had high biocompatibility, allowing the good growth of cells on the surface. The measurement of alkaline phosphatase activity and osteogenesis gene expression demonstrated that this composite system could promote the differentiation of MC3T3-E1 cells into osteoblasts. In addition, the *in vivo* cranial bone defect repair experiment confirmed that this composite hydrogel could promote the regeneration of new bones. In summary, Sr-HA/PTH_1-34_ composite hydrogel is a highly promising bone repair material.

## 1 Introduction

Bone tissue engineering has emerged as a promising strategy for addressing the limitations associated with traditional methods of bone repair and reconstruction. Current procedures often involve graft transplantation, which faces challenges such as donor site morbidity, limited graft availability, and potential immunogenic responses. As a result, there is a growing interest in developing alternative strategies that can stimulate the body’s innate regenerative capacity. Among these, *in-situ* bone tissue engineering stands out as it combines the principles of biology and engineering to create functional bone tissue directly at the site of injury or disease (Kim et al., 2011; Hou et al., 2018; Chen et al., 2021a; Chen et al., 2021b). The use of scaffolds that either have inherent bone-inducing properties or are functionalized with osteoinductive growth factors is increasingly being recognized as a key factor in successful *in-situ* bone regeneration (Hasani-Sadrabadi et al., 2020a; Wan et al., 2022; Zhang et al., 2023). Novel scaffold materials, including bioactive ceramics, synthetic polymers, and composite materials, have been engineered to not only provide structural support but also to facilitate bone cell adhesion, proliferation, differentiation, and ultimately, new bone formation (Annamalai et al., 2018; Johnson et al., 2019; Olov et al., 2022; Liu et al., 2023).

An injectable hydrogel has the advantages of high-water content, injectability, *in situ* formation, controllable physical and chemical properties, and high biocompatibility (Cui et al., 2019; Zhao et al., 2019; Hasani-Sadrabadi et al., 2020b; Wu et al., 2021). It has been considered one of the most promising biological materials and is used in the field of bone tissue engineering. Furthermore, the injectable hydrogel can mini-invasively load cells, drugs, and various biological factors and thus has several advantages in bone tissue engineering (Olov et al., 2022; Zhao et al., 2022). Therefore, using mini-invasive techniques for administering the injectable hydrogel loaded with bone regeneration–promoting factors into the high-risk regions of osteoporotic fractures in elderly people with initial fractures, followed by *in situ* solidification, can rapidly enhance the mechanical strength of local bone tissues and exert short-term protective effects (Zhang et al., 2020; Oprita et al., 2022). In addition, the controlled local release of biological factors and the degradation of the hydrogel can persistently enhance the bone density and bone mass of patients and thus exert long-term protective effects (Cheng et al., 2020). This treatment can be used to treat OP and prevent the occurrence of fractures in key regions.

In recent years, various types of gelatin hydrogels have been widely investigated and used for bone defect repair. Gelatin has several advantages, including high biocompatibility, biodegradability, low production cost, and capability of being chemically modified. For instance, gelatin methacryloyl (GelMA) hydrogel is a relatively ideal bone-substitute material. The methacrylic acid groups of GelMA are linked to the amino group on the side chain of gelatin, which can well mimic the extracellular matrix (ECM) (Chai et al., 2022; Wang et al., 2022). The chemical cross-linking of GelMA can effectively maintain cell viability, combine bioactive factors that promote cell or bone regeneration, and thus produce more excellent biological materials for bone tissue engineering (Yue et al., 2015). Heparin hydrogel is also a material widely used as a bone tissue engineering material, which has not only high histocompatibility but also high bioactivity (Sakiyama-Elbert, 2014; Nilasaroya et al., 2021). Combining chemically modified heparin with other high-molecular-weight materials can produce bone-substitute materials for bone defect repair. Strontium (Sr) is an extremely important element in the human skeleton (Place et al., 2011; Malinovsky et al., 2013). Various previous studies demonstrated that Sr could inhibit bone resorption to increase bone strength and improve the rate of the bone union. Sr ranelate is an effective drug for treating OP in clinical practice (Tournis et al., 2006). Recent studies have also demonstrated the osteogenic capability of Sr The supplementation of Sr ions to bone-substitute materials, such as bioactive glass and calcium phosphate, improves not only the mechanical properties of the materials but also the bone induction capability of the system, thus inducing the differentiation of osteoblasts *in vitro* and accelerating bone regeneration in the area of osteoporotic bone defect *in vivo* (Kargozar et al., 2019; Demirel and Kaya, 2020). Hydroxyapatite (HAP) is the major component of bones and teeth in vertebrates, which has high bioactivity and biocompatibility. Compared with conventional bone-substitute materials such as metals (e.g., stainless steel and titanium alloy) and ceramics (e.g., aluminum oxide and silicon nitride), HAP has the advantages of high resistance to corrosion and high capability of inducing bone generation (Shi et al., 2021). In addition, the degradation of HAP also eliminates the safety concerns associated with the use of conventional materials. Previous studies on the application of HAP as bone-substitute materials mainly focused on two aspects: HAP coating and human bone biomimetic regenerative material. Compared with HAP, strontium-substituted hydroxyapatite (Sr-HA) could more significantly accelerate bone union and enhance bone-forming capability (Vestermark, 2011). Small amounts of Sr-HA with the hydrogel also have such effects.

Achieving the balance between osteoblasts and osteoclasts is critical for effectively promoting bone regeneration. Currently, teriparatide (PTH_1-34_) is the only bone regeneration–promoting drug approved by the Food and Drug Administration (Gomes-Ferreira et al., 2022). The unique capability of PTH_1-34_ in activating osteoclasts and osteoblasts can help achieve the balance between bone resorption and bone regeneration and thus maintain the good state of bone. Various previous studies demonstrated that the local intermittent injection of PTH could effectively repair bone defects (Yoshida et al., 2019). However, the development of a local drug delivery system is generally limited, restricting the precise release of drugs. On the contrary, the local high-dose injection of PTH_1-34_ may induce net bone resorption (Chandra et al., 2014; Orbeanu et al., 2022). Therefore, we hypothesized that the local low-dose PTH_1-34_ intervention could provide a microenvironment to promote bone regeneration.

**FIGURE 1:**
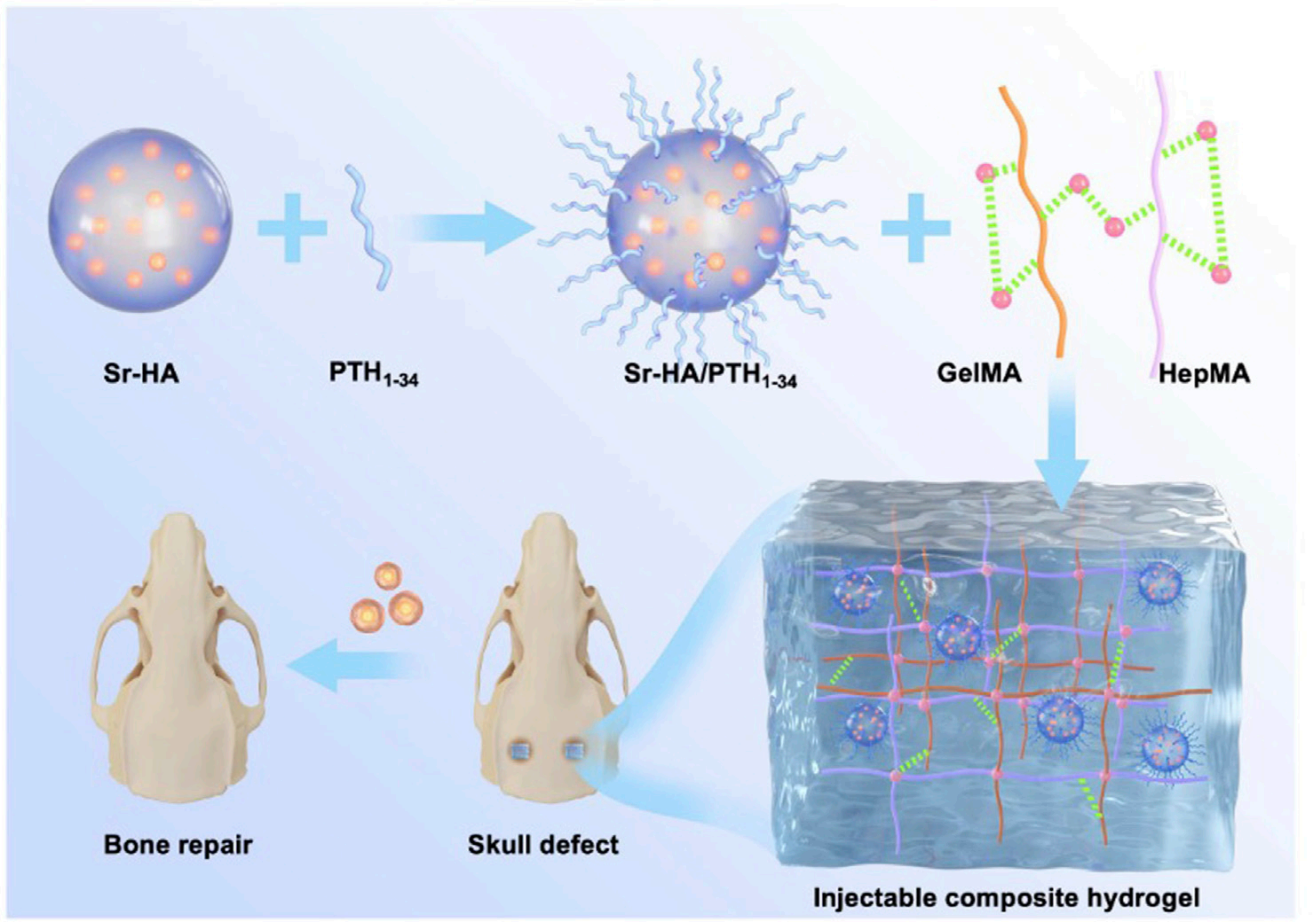
Schematic illustration of a composite hydrogel for local low dose delivery of PTH_1-34_ for bone repair.

In this study, Sr-HA was used to absorb PTH_1-34_ polypeptide and obtain Sr-HA/PTH_1-34_. Then, double-bonded heparin (HepMA) and methacrylate gelatin (GelMA) were used as the main body of the hydrogel to produce the precursor solution of the hydrogel with high biocompatibility. Further, Sr-HA/PTH_1-34_ was combined with the precursor solution of the hydrogel to prepare the Sr-HA/PTH_1-34_composite hydrogel, which could allow the local controlled release of low-dose PTH to achieve the repair of defected bone tissues. *In vitro* cellular experiments showed that Sr-HA/PTH_1-34_-loaded hydrogel system had high biocompatibility (Figure 1). This composite hydrogel could promote the differentiation of MC3T3-E1 cells into osteoblasts. *In vitro* cranial bone defect repair experiments confirmed that this composite hydrogel could effectively promote the regeneration of new bones. The composite hydrogel was mini-invasively injected into the site of osteoporotic fractures, followed by *in situ* solidification, which could rapidly enhance the mechanical strength of the local bone tissues and thus exert supporting effects. In addition, with the swelling and degradation of the composite hydrogel, the released PTH_1-34_ could induce bone marrow mesenchymal stem cells (BMSCs) to promote osteoblastic differentiation, persistently increase the bone density and bond mass at specific sites, and thus exert long-term protective effects.

## 2 Experimental

### 2.1 Preparation of the composite hydrogel

GelMA and HepMA were prepared as described in previous studies (Yue et al., 2015; Brown et al., 2017; Kurian et al., 2022). In brief, gelatin or heparin was dissolved in phosphate-buffered saline (PBS) to prepare a 10% gelatin solution or 2% heparin solution. Then, a certain amount of methacrylic anhydride was added to react for 3–4 h. A high amount of deionized water was added for dialysis for 5 days, which was then freeze-dried to obtain the GelMA and HepMA monomers. The prepared HepMA and GelMA were dissolved in PBS and mixed evenly. Sr-HAwas dissolved in a weak acid to prepare a homogeneous emulsion. The aforementioned solutions were mixed evenly, and then PTH_1-34_ was added. Blue ray photoinitiator Lithium phenyl-2,4,6-trimethylbenzoylphosphinate (LAP) was added to induce rapid cross-linking and solidification, forming the injectable composite hydrogel. LAP has high biocompatibility and can be used to induce the solidification of the hydrogel that wraps the cells (Wang et al., 2012; Magalhães et al., 2020).

### 2.2 Physical and chemical properties of the composite hydrogel

#### 2.2.1 Gel formation time of the hydrogel

The tube inversion method was used to measure the gel formation time of the hydrogel. Different concentrations of the hydrogel were prepared at 37°C. The gel formation time was the time during which the inverted tube showed no flow. All samples were prepared in triplicate for the measurement.

#### 2.2.2 Analysis of the microstructure of the hydrogel

Cylinders with a diameter of 6 mm and thickness of 1 mm were prepared, which were then freeze-dried. Then, the samples were placed on the platform of a scanning electron microscope. The sizes and numbers of pores, as well as the pokiness rate, were analyzed using scanning electron microscopy.

#### 2.2.3 Analysis of mechanical properties

The Shimadzu mechanical system (AG-IS, Shimadzu, Japan) was used to assess the mechanical properties of the hydrogel. The cylindrical hydrogel with a diameter of 8 mm and thickness of 6 mm was compressed at the strain rate of 5 mm/min, and the modulus of compression was calculated using the slope of the stress–strain curve in the range of linearity corresponding to 5%–10% of strain. The limiting stress was the highest stress before the rupture of the hydrogel.

#### 2.2.4 Analysis of the swelling behavior of the hydrogel

The hydrogel was freeze-dried, and the dry weight (*W*_d_) was measured. The dry hydrogel was placed in deionized water and retrieved at predefined time points (i.e., 0.5, 1, 3, 6, 12, 24, and 36 h). The wet weight (*W*_w_) of the hydrogel at each time point was measured. The swelling ratio was calculated using the equation: swelling ratio = (*W*_w_–*W*_d_)/*W*_d_ × 100%, where *W*_w_ is the wet weight at predefined time points and *W*_d_ is the dry weight of the initial material.

#### 2.2.5 Analysis of the degradation of the hydrogel

The hydrogel was freeze-dried, and the dry weight (*W*_d_) was measured. The hydrogel was placed in 1× PBS, and the PBS was changed every other day. The hydrogel was retrieved on a predefined day 7 and freeze-dried, and the dry weight (*W*_t_) was measured. The hydrogel was then placed in 1× PBS again and retrieved seven more days later (a total of 14 days) and freeze-dried, and the dry weight was measured. The experiment was continued for 28 days. The remaining mass of the hydrogel was calculated using the equation: Remaining mass = *W*_t_/*W*_d_ × 100%, where *W*_t_ is the dry weight at the predefined time points and *W*_d_ is the initial dry weight.

### 2.3 *In vitro* drug release test of the composite hydrogel

The drug release test of the hydrogel was performed. In brief, 1 mL of the hydrogel sample loaded with PTH_1-34_ (1 mg/mL) was dispersed in 5 mL of 1× PBS and vortexed in a water bath at 37°C. The samples were retrieved at different time points (i.e., 0, 5, 10, 20, 30, 50, 70, 90, 120, 140, and 180 h) and centrifuged to obtain the supernatant. Then, high-performance liquid chromatography was used to measure the PTH_1-34_ level in the solution. The cumulative drug release level was assessed, and the cumulative drug release curve was plotted. The chromatographic column used in this study was the X-Bridge-Shield-RP-C18 column (Waters, United States) (50 × 4.6 mm^2^, 3.5 µm), the flow rate was 2.5 mL/min, and the column temperature was 40°C. The volume of the sample load was 100 μL, and the wavelength of measurement was 245 nm. The mobile phase A was 0.05% Trifluoroacetic acid (TFA)–water solution and the mobile phase B was 0.05% TFA–acetonitrile, which were used for gradient elution.

### 2.4 *In vitro* assay of the composite hydrogel

#### 2.4.1 Biocompatibility of the composite hydrogel

MC3T3-E1 (ATCC, United States) were cultured in α-minimum essential medium (α-MEM, Gibco, United States) supplemented with 10% fetal bovine serum (Gibco), 1% penicillin, and streptomycin (Gibco). The cells were cultured under standard conditions in the presence of 5% CO_2_ and at 95% humidity. The merged cells were collected after digestion with EDTA containing 0.25% trypsin (Gibco) for the subsequent experiments.

The live/dead detection reagent Calcein AM and propidium (PI) were prepared, added to cells co-cultured with the hydrogel, and incubated at 37°C for 15 min. The samples were washed with PBS twice, and then an inverted fluorescence microscope was used for imaging. The CellTiter 96 aqueous solution cell proliferation assay (MTS, Promega, United States) was used to measure the viability of hBMSCs. The culture medium was discarded after the cells were cultured for 1, 3, and 7 days, and then 200 μL of the fresh culture medium with 10% MTS was added to incubate the cells for 4 h. A microplate reader (ELX808, BioTek, United States) was used to measure the optical density at 490 nm.

#### 2.4.2 Induction of osteoblast differentiation by the hydrogel

The alkaline phosphatase (ALP) activity was measured to assess osteoblast differentiation. The BCIP/NBT ALP detection kit (Beyotime, China) was used to measure the ALP activity following the manufacturer’s protocols. In brief, the 48-well plate was fixed with formalin solution, washed with PBS, and stained with 300 μL of the dye. Then, the cells were rinsed with running water and blotted dry, and the Olympus CKX53 microscope was used for imaging.

#### 2.4.3 Expression of osteogenesis-related genes induced by the composite hydrogel

Quantitative real-time polymerase chain reaction (qRT-PCR) was performed to assess the expression of osteogenesis-related genes. The AG RNAex Pro reagent (AG21102, Accurate Biotechnology, China) was used to extract the total RNA from cells following the manufacturer’s protocols. The Nanodrop 2000 spectrophotometer (Thermo Scientific, United States) was used to quantitatively assess the RNA level, and the OD260/OD280 was between 1.9 and 2.1. Total RNA (1 μg) was obtained and reverse transcribed to obtain cDNA using the PrimeScript RT kit (RR037A, TaKaRa, Japan). The StepOnePlus quantitative PCR system (Applied Biosystems, United States) with TB Green pre-mixed Ex Taq (RR420A, Takara, Japan) was used for the qRT-PCR. When assessing osteogenesis-related genes (*ALP*, osteocalcin (*OCN*), osteopontin (*OPN*), and runt-related transcription factor 2 (*RUNX2*)), GAPDH was used as the internal reference, and the 2^−ΔΔCT^ method was used to estimate the relative expression of mRNA. All the primers were synthesized by Sangon Biotech, China.

### 2.5 Induction of the cranial bone defect model

All the animal procedures were approved by the Animal Research Committee of Shenzhen People’s Hospital, Jinan University. Forty-eight male rats (10 weeks old, body weight of 280–300 g) were obtained from the Guangzhou Animal Experiment Center. After the rats were anesthetized with an intraperitoneal injection of pentobarbital sodium (45 mg/kg), a manual electric trephine was used to make two symmetric full-thickness round defects of 4.5-mm diameter on the bilateral sides of the skull in the rats. GelMA, GelMA + HepMA + Sr-HA, and GelMA + HepMA + Sr-HA + PTH_1-34_ were injected into the skull defects (each group consisting of six rats), and normal saline was injected into the rats in the control group (sham surgery group). The antibiotics were injected into all the rats after the surgery once every 3 days. All the rats were sacrificed by cervical dislocation after 4 weeks. The skull was obtained and stored in 4% formalin, which was consequently used for imaging and histological analyses.

### 2.6 Micro-CT assay

The rats were sacrificed 4 weeks after the surgery, the root skull was collected and fixed in 4% paraformaldehyde (PFA), and then the skull was scanned using the high-resolution images were acquired using the 3D creator software, and then the parameters including bone mass density (BMD), bone volume (BV/TV), bone trabecular number (Tb.N), and trabecular separation (Tb. sp) were calculated.

### 2.7 Histological analysis and immunohistochemistry

After micro-CT assay, the skull was immersed in 10% EDTA for decalcification, followed by dehydration and paraffin embedding. Then, 5-µm-thick slices were prepared, stained using hematoxylin and eosin (H&E) (Sigma, United States) and Masson trichrome staining (Solarbio, China) for assessing the histological morphology of the defect region of the skull. For the immunohistochemistry (IHC), the skull was fixed in 4% PFA, dehydrated, and paraffin embedded. The slices (5 µm) were prepared and dewaxed with xylene, rehydrated with ethanol, processed using 3% H_2_O_2_ in absolute methanol for 30 min, immersed and boiled in citrate buffer (pH = 6.0) for 10 min, and then cooled to room temperature. The slices were blocked with normal goat serum for 30 min and then incubated with rat anti-RUNX2 and anti-OCN monoclonal antibodies at 4°C overnight. Afterward, the samples were subjected to processing using an Avidin-Biotin Complex (ABC) kit (Zhongshanjinqiao Biotechnology Co., Ltd, China) (biotin: 1 h; streptavidin: 30 min, 37°C) and then incubated using the 3,3′Diaminobenzidine (DAB) kit (Zhongshanjinqiao Biotechnology Co., Ltd.) for 1 min. The Mayer hematoxylin was used for counterstaining. The omission of the primary antibody was used as the negative control.

### 2.8 Statistical analysis

All the data were described as means and standard deviations (≥3 samples). One-way analysis of variance and paired-sample *t*-test were used for the statistical analysis. A *p*-value < 0.05 indicated a statistically significant difference. All the statistical analysis and figure plotting were performed using the Origin 8.5 software.

## 3 Results and discussion

### 3.1 Preparation of the composite hydrogel and analysis of physical and chemical properties

**FIGURE 2:**
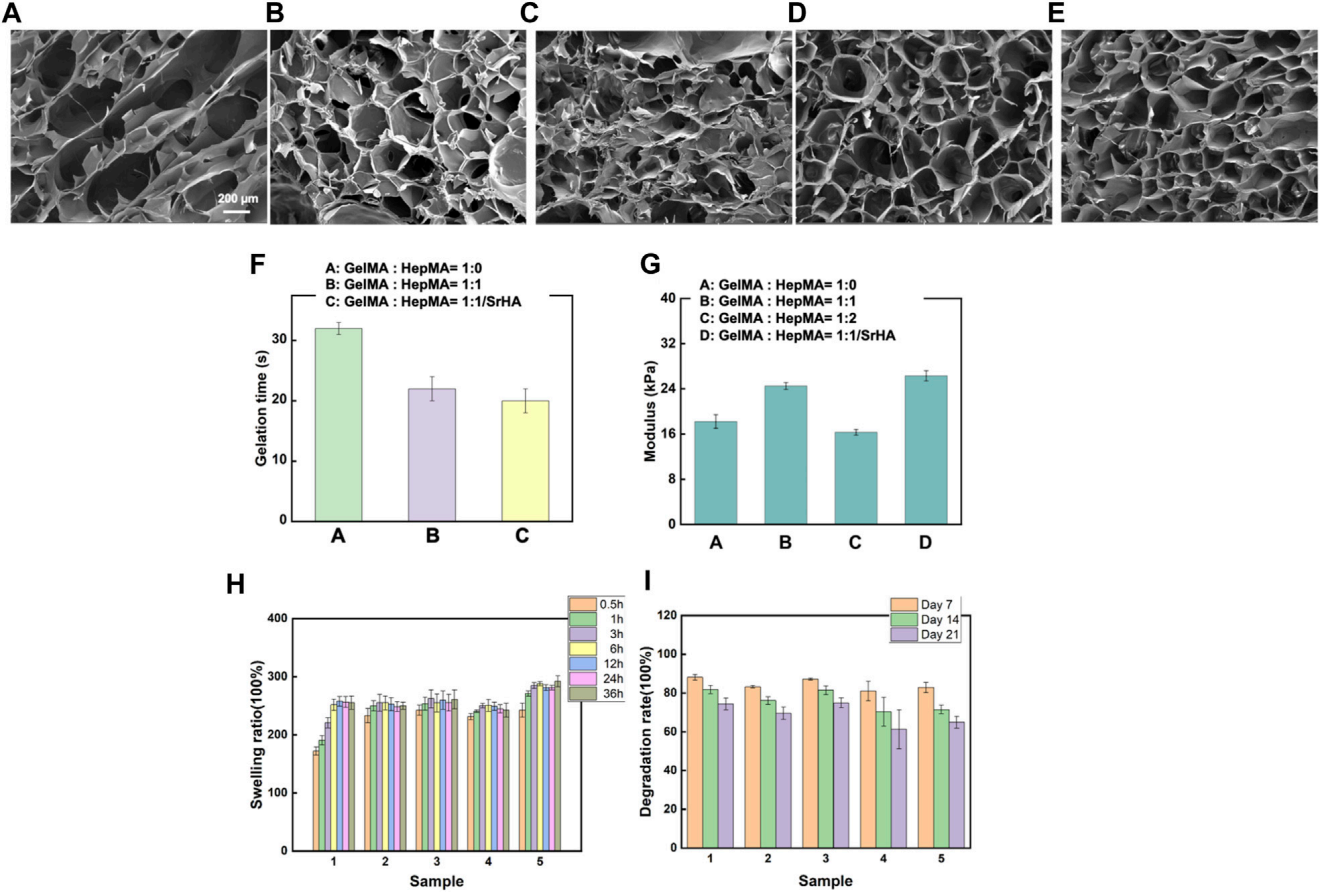
Physical and chemical properties of different hydrogels. **(A–E)** Microstructural properties of the hydrogels **(A)**: GelMA hydrogel; **(B)** SrHA; **(C)** GelMA + HepMA hydrogel; **(D)** GelMA + HepMA + SrHA hydrogel; **(E)** GelMA + HepMA + SrHA/PTH_1-34_ hydrogel). **(F)** Time of gel formation of the hydrogel. **(G)** Comparison of modulus of compression of different hydrogel preparations. **(H, I)** Swelling and degradation behaviors of different hydrogel systems.

The composite hydrogel system was prepared by rapid solidification of HepMA (10 wt%) and GelMA (2 wt%) induced by a blue ray photoinitiator LAP. The gel formation time of the different systems is shown in Supplementary Table S1. The Sr-HA showed no significant influence on the gel formation time of the ultimate systems (Figure 2F). The porosity and sizes of pores were critical for some cellular functions, such as influencing cell growth and nutrient transportation. The freeze-dried samples were scanned using electron microscopy. The findings are shown in Figures 2A–E. Adding HepMA to GelMA could increase the cross-linking of the hydrogel and the number of pores in the hydrogel, making the network more dense and even. In addition, when the ratio of GelMA to HepMA was 1:1, the best modulus of compression was obtained. The introduction of HAP could reduce the modulus of compression of the hydrogel (Figure 2G). The swelling equilibrium of the hydrogel was achieved in approximately 12 h. Compared with pure GelMA hydrogel, adding HepMA reduced the swelling ratio of the system, and adding SrHA also slightly but not significantly reduced the swelling ratio (Figure 2H). After the different hydrogel preparations were freeze-dried, the degradation assay was performed by immersing the samples in PBS, followed by degradation after 7, 14, and 21 days. The findings are shown in Figure 2I, which indicated that the introduction of Sr-HA could delay the degradation of the system.

### 3.2 *In vitro* drug release assay of the composite hydrogel

**FIGURE 3:**
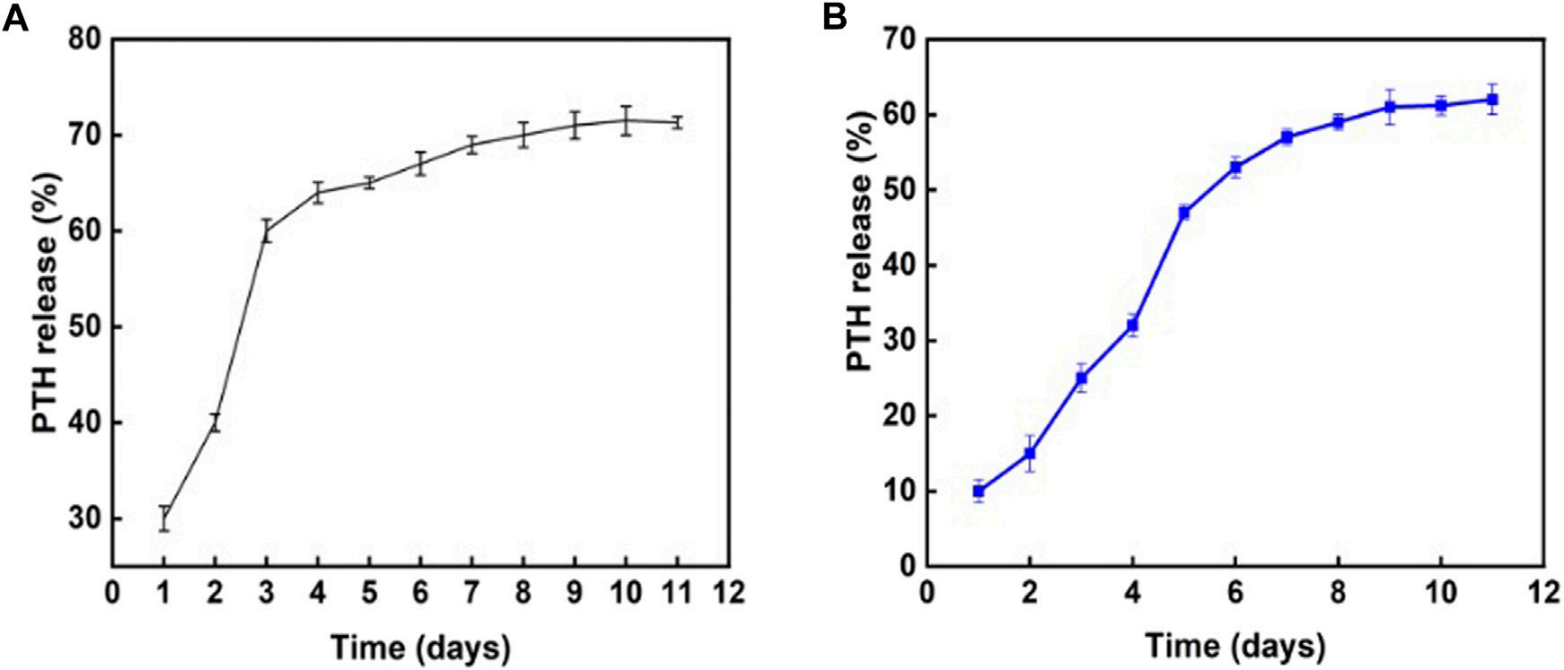
Influence of different hydrogel systems on PTH_1-34_ release behaviors. **(A)** PTH_1-34_ mixed with the hydrogel system of GelMA:HepMA = 1:1. **(B)** PTH_1-34_ mixed with the hydrogel system of GelMA:HepMA = 1:1 (with SrHA).

The *in vitro* effects of the composite hydrogel on PTH_1-34_ release were assessed. As shown in Figure 3A, the direct combination of PTH_1-34_ with the hydrogel resulted in a relatively fast release in the first 3 days. On the contrary, introducing Sr-HA into loaded PTH_1-34_ reduced the release rate in the initial stage and allowed the slow, low-dose release of PTH_1-34_ in the following week (Figure 3B).

### 3.3 *In vitro* cellular experiments of the hydrogel

**FIGURE 4:**
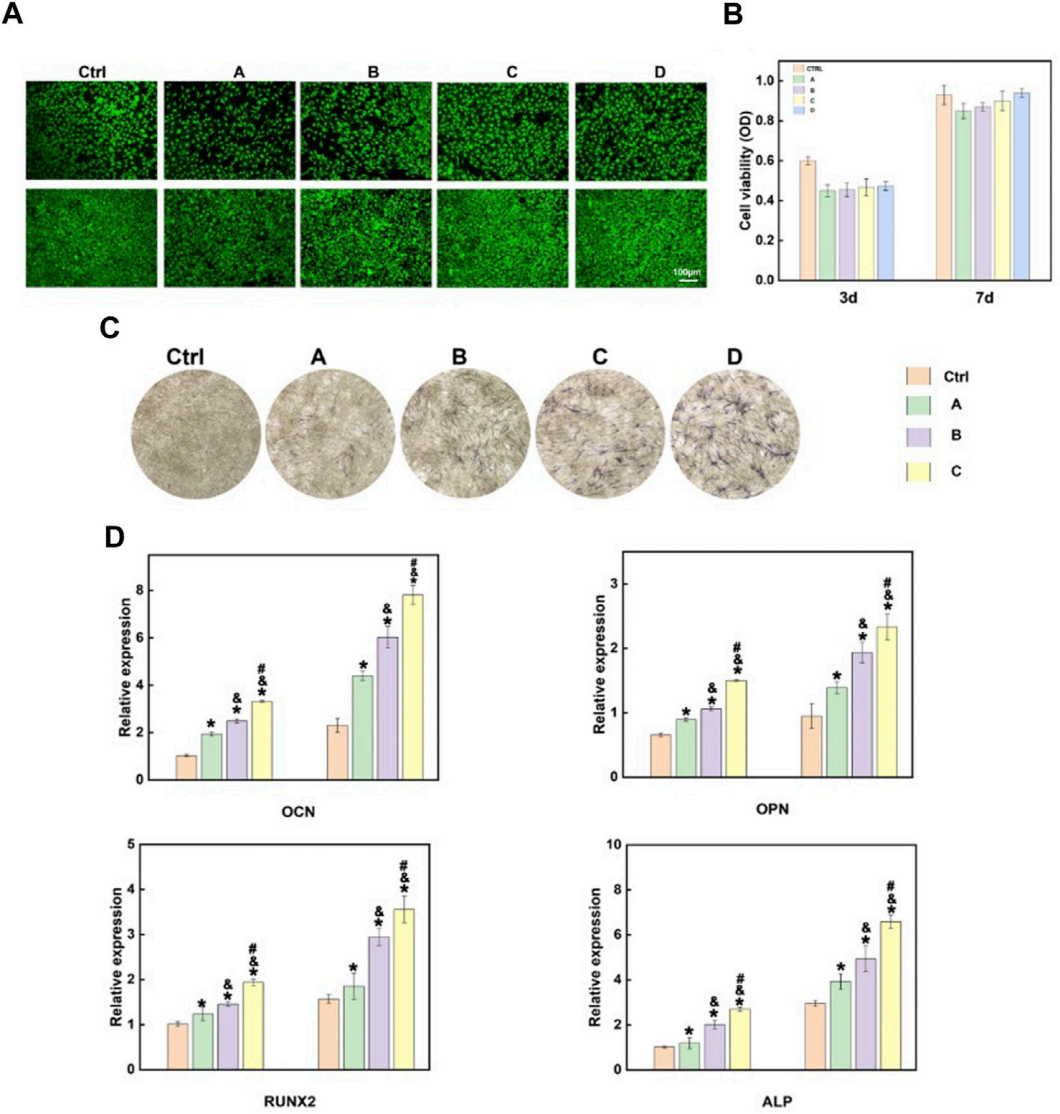
*In vitro* cellular experiments of the composite hydrogel. **(A)** Live-dead cell staining findings after MC3T3-E1 cells were co-cultured with different hydrogel systems for 3 and 7 days **(B)** Quantitative analysis of the cell proliferation of MC3T3-E1 cells co-cultured with different hydrogel systems for 3 and 7 days using MTS. **(C)** The ALP activity of MC3T3-E1 cells after 7-day growth on various hydrogels-conditioned medium **(A)**: GelMA hydrogel; **(B)**GelMA + HepMA hydrogel; **(C)** GelMA + HepMA + SrHA hydrogel; **(D)** GelMA + HepMA + SrHA/PTH_1-34_ hydrogel); **(D)** Measurement of the expression of different osteogenesis-related genes **(A)**:GelMA + HepMA hydrogel; **(B)** GelMA + HepMA + SrHA hydrogel; **(C)** GelMA + HepMA + SrHA/PTH_1-34_ hydrogel).

MC3T3-E1 cells were co-cultured with the hydrogel, and MTS and live-dead cell staining were performed after the cells were cultured for 3 and 7 days, respectively, to assess the influences of different hydrogel systems on cell viability. As shown in Figures 4A, B, the co-culture of cells with different hydrogel systems-maintained cell viability. In addition, the introduction of Sr-HA and PTH_1-34_promoted the proliferation of cells. Scanning electron microscope (SEM) was used to visualize the state of cells co-cultured with the hydrogel (Supplementary Figure S1), showing that the cells could attach to the surface of hydrogel materials and survive. ALP staining was performed to assess the effects of the hydrogel systems on the osteoblast differentiation of MC3T3-E1 cells. The findings showed that compared with the control group, the hydrogel loaded with Sr-HA and PTH_1-34_ showed the most significant ALP staining signals, which were higher than those for the system loaded with Sr-HA only, indicating that Sr-HA and PTH_1-34_ could jointly promote the differentiation of MC3T3-E1 cells into osteoblasts (Figure 4C). The hydrogel systems loaded with Sr-HA increased the expression of osteogenesis-related genes, including OCN, OPN, RUNX2, and ALP (Figure 4D). More significantly, the simultaneous loading of Sr-HA and PTH in the system resulted in the highest expression levels of the osteogenesis-related genes. These findings demonstrated that the joint effects of Sr-HA and PTH_1-34_ could better promote the differentiation into osteoblasts.

### 3.4 *In vivo* effects of the composite hydrogel in promoting bone regeneration

**FIGURE 5:**
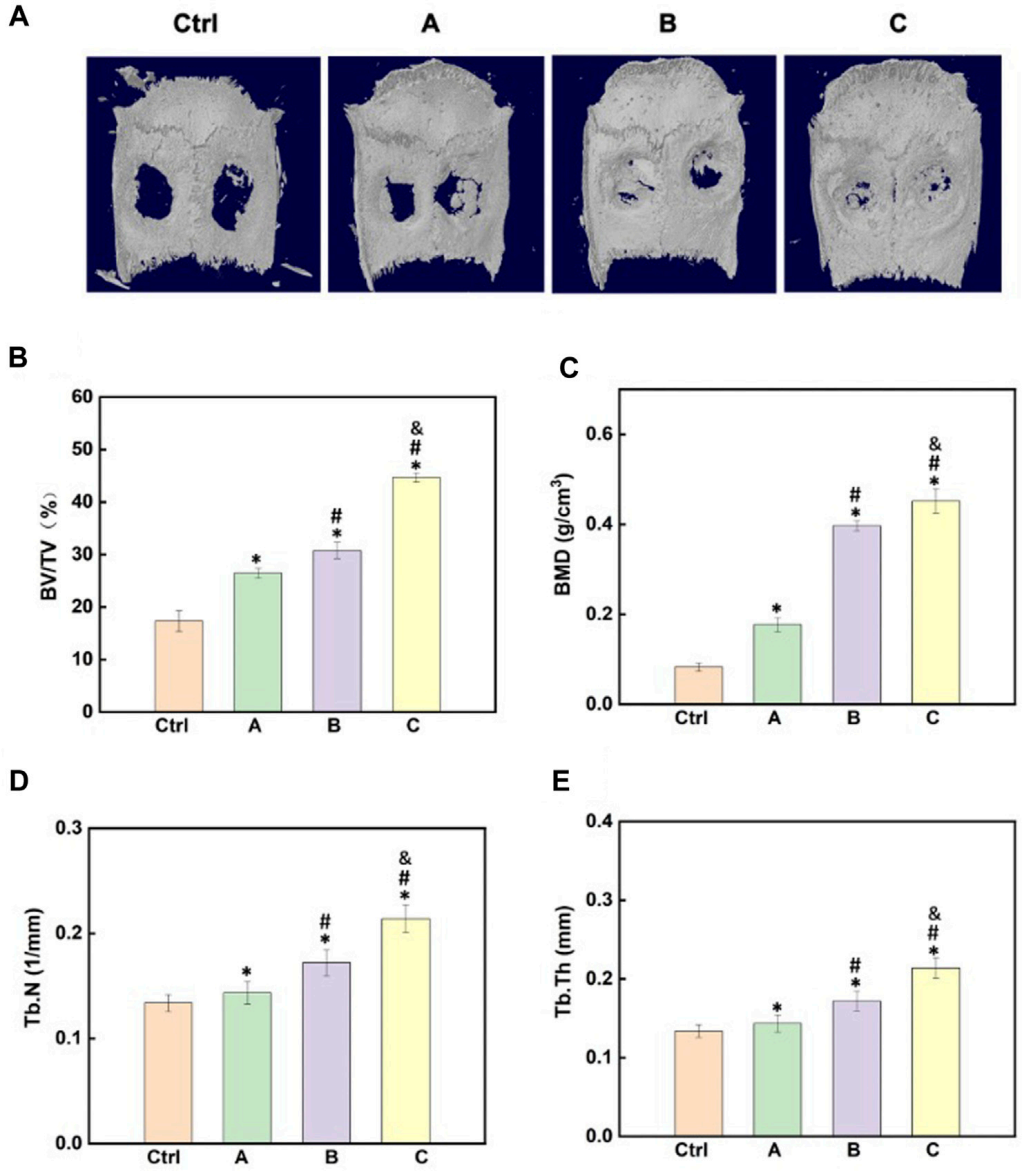
Effects of the composite hydrogel systems in promoting bone regeneration *in vivo.* **(A)** 3D reconstruction images of the skull after different hydrogel materials were implanted to the site of skull bone defects for 4 weeks. **(B–E)** Quantitative analysis of the micro-CT images for the assessment of BV/TV, BMD, Tb.N, and Tb.Th. **(A)**: GelMA + HepMA hydrogel; **(B)** GelMA + HepMA + SrHA hydrogel; **(C)** GelMA + HepMA + SrHA/PTH_1-34_ hydrogel).

After the materials were implanted in the body for 4 weeks, micro-CT images clearly displayed the differences in the regeneration of new bone tissues after treatment with different components (Figure 5). The images clearly showed that the *in vivo* osteogenesis effects were in agreement with the *in vitro* findings, and the hydrogel with both Sr-HA and PTH could better promote the repair and regeneration of bone tissues at the bone defects (Figure 5A). The quantitative analysis of BMD, BV/TV, Tb.N, and Tb.Th was performed to further assess the osteogenesis effects. The findings showed that after treatment with the composite hydrogel system for 4 weeks, the hydrogel loaded with both Sr-HA and PTH could induce the regeneration of new bones at the site of bone defects, and the effects were higher than those for the hydrogel system loaded with Sr-HA only (Figures 5B–E).

### 3.5 Histological assessment and IHC of the new bones

**FIGURE 6:**
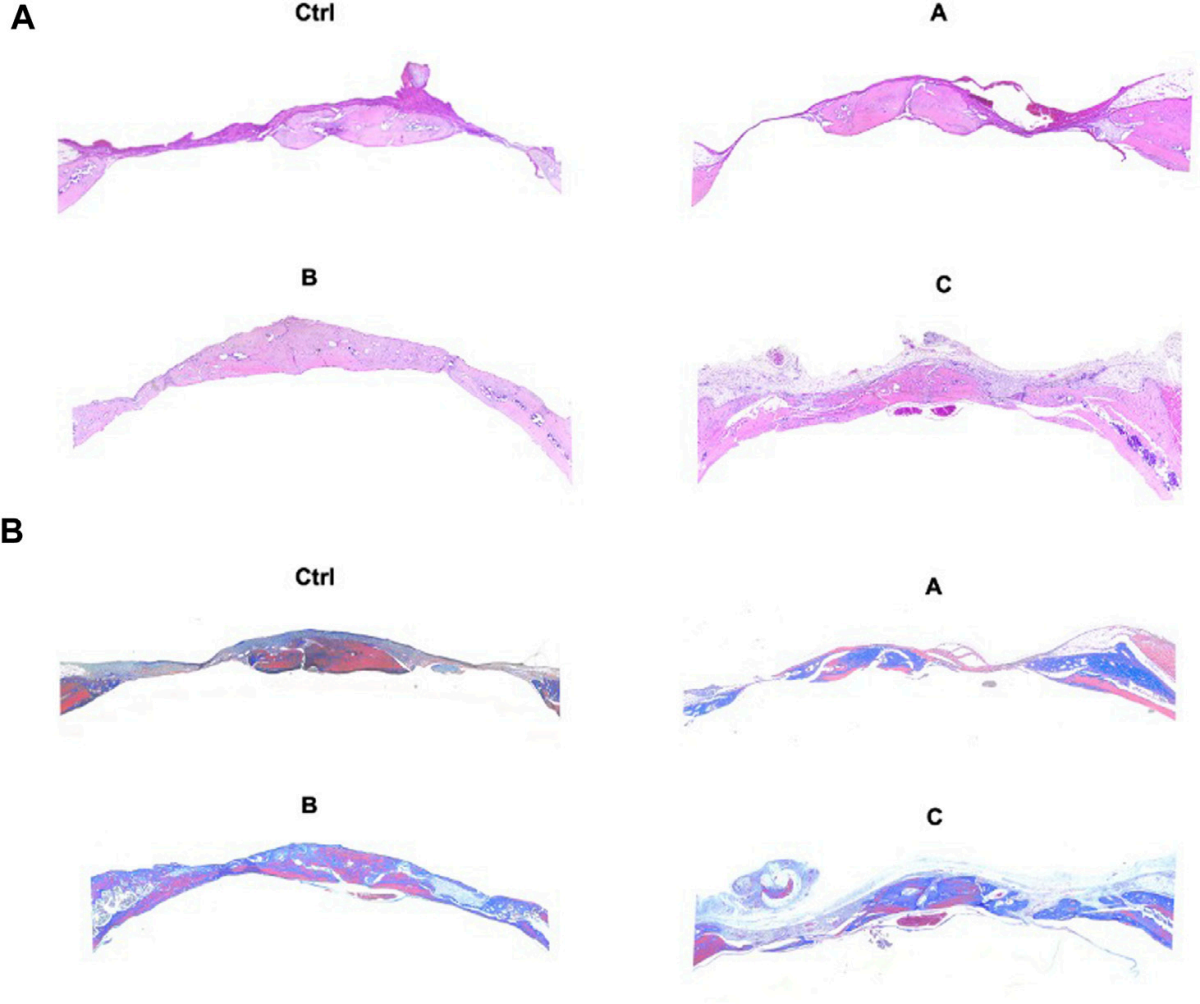
H&E staining **(A)** and Masson trichrome staining **(B)** after the bone defects were treated with different hydrogel systems for 4 weeks (4×).

**FIGURE 7:**
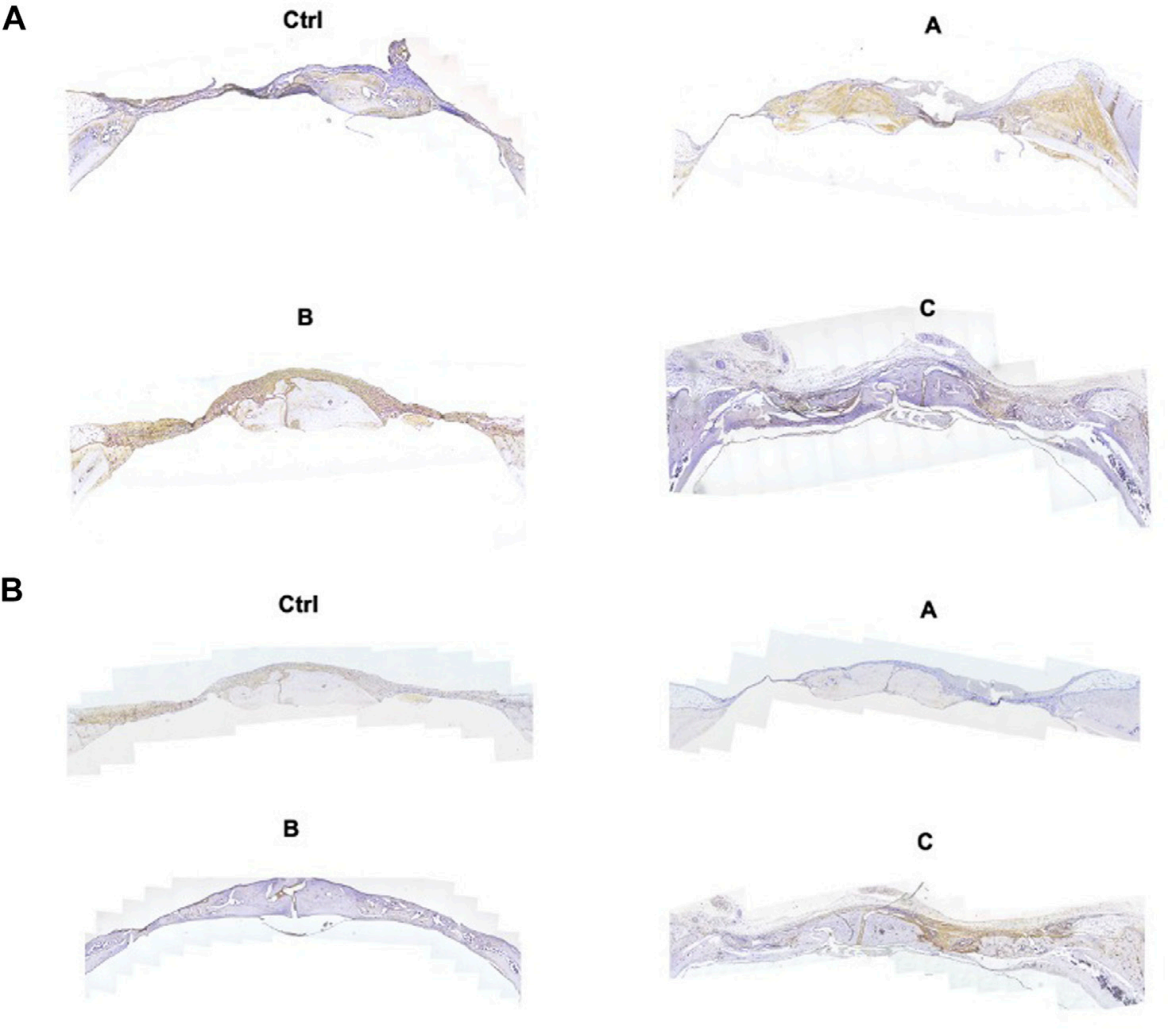
IHC images of OCN **(A)** and RUNX2 **(B)** after the bone defects were treated with different hydrogel systems for 4 weeks (4×).

H&E staining, Masson trichrome staining, and IHC were used for the assessment of bone regeneration at the site of bone defects to further verify the effects of the composite hydrogel systems on bone regeneration. The Figure 6 shows the H&E staining and Masson trichrome staining results of bone defects in different groups. The findings were in agreement with the micro-CT results. Compared with other treatments, Sr-HA and PTH co-treatment induced the highest amount of new bone tissues, with the new bones filling the whole defect site, and the regenerated collagenous fibers were denser than those in other groups. The Masson trichrome staining further showed that the GelMA/HepMA/SrHA/PTH group had more calcified bone-like tissues connected to each other, indicating the presence of more new bones with higher maturity. The IHC assay showed slightly lower expression in the GelMA/HepMA/Sr-HA group, while the expression of OCN and RUNX2 significantly increased in the GelMA/HepMA/SrHA/PTH group (Figures 7A, B).

## 4 Conclusion

In this study, we established a composite hydrogel capable of the controlled release of PTH to achieve the local controlled release of low-dose PTH, providing a good microenvironment for promoting bone regeneration at the site of bone defects. First, Sr-HA was used to absorb the PTH polypeptide, which was then mixed with the precursor solution of GelMA/HepMA hydrogel to prepare the composite hydrogel system with high mechanical strength and high biocompatibility and capable of the local controlled release of low-dose PTH by light cross-linking. The treatment of bone defects using the composite hydrogel could effectively promote the cells to differentiate into osteoblasts and promote tissue regeneration via the local controlled release of low-dose PTH. In addition, the mini-invasive injection of the composite hydrogel into the site of osteoporotic fracture, followed by *in situ* solidification, could rapidly enhance the mechanical strength of local bone tissues, thus exerting the supporting effects. Furthermore, with the swelling and degradation of the hydrogel, the PTH released by the composite hydrogel could induce the BMSCs to promote bone differentiation, persistently improve the local bone density and bone mass, and thus exert long-term protective effects.

Despite the promising results demonstrated in our research, several limitations must be acknowledged. Firstly, the results indicated that the local low dose PTH treatment could better activate the signaling pathways associated with osteoblasts and thus promote bone regeneration. However, the exact mechanisms involved in the effects on osteoblasts and osteoclasts were not investigated, which should be explored in future. Secondly, the current study only examined the short-term effects of the developed hydrogel in bone repair. Long-term studies are necessary to assess the persistence of the newly formed bone and the possible long-term effects or complications related to the hydrogel or its degradation products. In addition, the use of strontium and PTH was based on their proven benefits in osteoporosis treatment and bone regeneration. However, the specific doses and ratios used in this study were determined empirically. Further dose-response studies may be necessary to optimize the concentrations of strontium and PTH for maximal therapeutic effects and minimal side effects. Nevertheless, future work will need to explore the long-term safety, efficacy, and practicality of these hydrogel systems.

## Data Availability

The original contributions presented in the study are included in the article/Supplementary Material, further inquiries can be directed to the corresponding author.

## References

[B1] Annamalai R. T. Turner P. A. Carson IV W. F. Levi B. Kunkel S. Stegemann J. P. (2018). Harnessing macrophage-mediated degradation of gelatin microspheres for spatiotemporal control of BMP2 release. Biomaterials 161, 216–227. 10.1016/j.biomaterials.2018.01.040 29421557 PMC5831261

[B2] Brown G. C. Lim K. S. Farrugia B. L. Hooper G. J. Woodfield T. B. (2017). Covalent incorporation of heparin improves chondrogenesis in photocurable gelatin-methacryloyl hydrogels. Macromol. Biosci. 17 (12), 1700158. 10.1002/mabi.201700158 29068543

[B3] Chai S. Huang J. Mahmut A. Wang B. Yao Y. Zhang X. (2022). Injectable photo-crosslinked bioactive BMSCs-BMP2-GelMA scaffolds for bone defect repair. Front. Bioeng. Biotechnol. 10, 875363. 10.3389/fbioe.2022.875363 35402421 PMC8989181

[B4] Chandra A. Lin T. Tribble M. B. Zhu J. Altman A. R. Tseng W.-J. (2014). PTH1–34 alleviates radiotherapy-induced local bone loss by improving osteoblast and osteocyte survival. Bone 67, 33–40. 10.1016/j.bone.2014.06.030 24998454 PMC4154509

[B5] Chen X. Tan B. Bao Z. Wang S. Tang R. Wang Z. (2021a). Enhanced bone regeneration via spatiotemporal and controlled delivery of a genetically engineered BMP-2 in a composite Hydrogel. Biomaterials 277, 121117. 10.1016/j.biomaterials.2021.121117 34517277

[B6] Chen X. Tan B. Wang S. Tang R. Bao Z. Chen G. (2021b). Rationally designed protein cross-linked hydrogel for bone regeneration via synergistic release of magnesium and zinc ions. Biomaterials 274, 120895. 10.1016/j.biomaterials.2021.120895 34020269

[B7] Cheng W. Ding Z. Zheng X. Lu Q. Kong X. Zhou X. (2020). Injectable hydrogel systems with multiple biophysical and biochemical cues for bone regeneration. Biomater. Sci. 8 (9), 2537–2548. 10.1039/d0bm00104j 32215404 PMC7204512

[B8] Cui Z.-K. Kim S. Baljon J. J. Wu B. M. Aghaloo T. Lee M. (2019). Microporous methacrylated glycol chitosan-montmorillonite nanocomposite hydrogel for bone tissue engineering. Nat. Commun. 10 (1), 3523–3533. 10.1038/s41467-019-11511-3 31388014 PMC6684526

[B9] Demirel M. Kaya A. I. (2020). Effect of strontium-containing compounds on bone grafts. J. Mater Sci. 55 (15), 6305–6329. 10.1007/s10853-020-04451-7

[B10] Gomes-Ferreira P. H. S. Micheletti C. Frigério P. B. de Souza Batista F. R. Monteiro N. G. Bim-Júnior O. (2022). PTH 1-34-functionalized bioactive glass improves peri-implant bone repair in orchiectomies rats: Microscale and ultrastructural evaluation. Mat. Sci. Eng. C 134, 112688. 10.1016/j.msec.2022.112688 35581064

[B11] Hasani-Sadrabadi M. M. Sarrion P. Pouraghaei S. Chau Y. Ansari S. Li S. (2020a). An engineered cell-laden adhesive hydrogel promotes craniofacial bone tissue regeneration in rats. Sci. Transl. Med. 12 (534), eaay6853. 10.1126/scitranslmed.aay6853 32161103

[B12] Hasani-Sadrabadi M. M. Sarrion P. Pouraghaei S. Chau Y. Ansari S. Li S. (2020b). An engineered cell-laden adhesive hydrogel promotes craniofacial bone tissue regeneration in rats. Sci. Transl. Med. 12 (534), eaay6853. 10.1126/scitranslmed.aay6853 32161103

[B13] Hou S. Lake R. Park S. Edwards S. Jones C. Jeong K. J. (2018). Injectable macroporous hydrogel formed by enzymatic cross-linking of gelatin microgels. ACS Appl. bio Mater. 1 (5), 1430–1439. 10.1021/acsabm.8b00380 PMC6836686 31701093

[B14] Johnson C. T. Sok M. C. P. Martin K. E. Kalelkar P. P. Caplin J. D. Botchwey E. A. (2019). Lysostaphin and BMP-2 co-delivery reduces *S. aureus* infection and regenerates critical-sized segmental bone defects. Sci. Adv. 5 (5), eaaw1228. 10.1126/sciadv.aaw1228 31114804 PMC6524983

[B15] Kargozar S. Montazerian M. Fiume E. Baino F. (2019). Multiple and promising applications of strontium (Sr)-containing bioactive glasses in bone tissue engineering. Front. Bioeng. Biotechnol. 7, 161. 10.3389/fbioe.2019.00161 31334228 PMC6625228

[B16] Kim S. Tsao H. Kang Y. Young D. A. Sen M. Wenke J. C. (2011). *In vitro* evaluation of an injectable chitosan gel for sustained local delivery of BMP‐2 for osteoblastic differentiation. J. Biomed. Mater. Res. Part B Appl. Biomaterials 99 (2), 380–390. 10.1002/jbm.b.31909 21905214

[B17] Kurian A. G. Singh R. K. Patel K. D. Lee J.-H. Kim H.-W. (2022). Multifunctional GelMA platforms with nanomaterials for advanced tissue therapeutics. Bioact. Mat. 8, 267–295. 10.1016/j.bioactmat.2021.06.027 PMC8424393 34541401

[B18] Liu Z. Zhang J. Fu C. Ding J. (2023). Osteoimmunity-regulating biomaterials promote bone regeneration. AJPS 18 (1), 100774. 10.1016/j.ajps.2023.100774 36751654 PMC9894904

[B19] Magalhães L. S. S. Santos F. E. P. Elias C. d. M. V. Afewerki S. Sousa G. F. Furtado A. S. (2020). Printing 3D hydrogel structures employing low-cost stereolithography technology. J. Funct. Biomater. 11 (1), 12. 10.3390/jfb11010012 32098431 PMC7151634

[B20] Malinovsky G. Yarmoshenko I. Zhukovsky M. Starichenko V. Modorov M. (2013). Strontium biokinetic model for mouse-like rodent. J. Environ. Radioact. 118, 57–63. 10.1016/j.jenvrad.2012.11.003 23246589

[B21] Nilasaroya A. Kop A. M. Morrison D. A. (2021). Heparin‐functionalized hydrogels as growth factor‐signaling substrates. J. Biomed. Mater Res. A 109 (3), 374–384. 10.1002/jbm.a.37030 32515102

[B22] Olov N. Bagheri-Khoulenjani S. Mirzadeh H. (2022). Injectable hydrogels for bone and cartilage tissue engineering: A review. Prog. Biomater. 11, 113–135. 10.1007/s40204-022-00185-8 35420394 PMC9156638

[B23] Oprita E. I. Iosageanu A. Craciunescu O. (2022). Progress in composite hydrogels and scaffolds enriched with icariin for osteochondral defect healing. Gels 8 (10), 648. 10.3390/gels8100648 36286148 PMC9602414

[B24] Orbeanu V. Haragus H. Crisan D. Cirstoiu C. Ristic B. Jamieson V. (2022). Novel parathyroid hormone-based bone graft, KUR-113, in treatment of acute open tibial shaft fracture: A phase-2 randomized controlled trial. J. Bone Jt. Surg. Am. 104 (5), 441–450. 10.2106/jbjs.20.02109 34971551

[B25] Place E. S. Rojo L. Gentleman E. Sardinha J. P. Stevens M. M. (2011). Strontium-and zinc-alginate hydrogels for bone tissue engineering. Tissue Eng. Part A 17 (21-22), 2713–2722. 10.1089/ten.tea.2011.0059 21682547

[B26] Sakiyama-Elbert S. E. (2014). Incorporation of heparin into biomaterials. Acta Biomater. 10 (4), 1581–1587. 10.1016/j.actbio.2013.08.045 24021232 PMC3949739

[B27] Shi H. Zhou Z. Li W. Fan Y. Li Z. Wei J. (2021). Hydroxyapatite based materials for bone tissue engineering: A brief and comprehensive introduction. Crystals 11 (2), 149. 10.3390/cryst11020149

[B28] Tournis S. Economopoulos D. Lyritis G. (2006). Strontium ranelate: A novel treatment in postmenopausal osteoporosis. Ann. N. Y. Acad. Sci. 1092 (1), 403–407. 10.1196/annals.1365.037 17308165

[B29] Vestermark M. T. (2011). Strontium in the bone-implant interface. Dan. Med. Bull. 58 (5), B4286. 10.1016/j.jpainsymman.2010.07.016 21535993

[B30] Wan X. Zhao Y. Li Z. Li L. (2022). Emerging polymeric electrospun fibers: From structural diversity to application in flexible bioelectronics and tissue engineering. Exploration 2, 20210029. 10.1002/exp.20210029 37324581 PMC10191062

[B31] Wang T. Nie J. Yang D. (2012). Dextran and gelatin based photocrosslinkable tissue adhesive. Carbohydr. Polym. 90 (4), 1428–1436. 10.1016/j.carbpol.2012.07.011 22944399

[B32] Wang T. Xu W. Zhao X. Bai B. Hua Y. Tang J. (2022). Repair of osteochondral defects mediated by double-layer scaffolds with natural osteochondral-biomimetic microenvironment and interface. Mater. today bio 14, 100234. 10.1016/j.mtbio.2022.100234 PMC8924418 35308043

[B33] Wu D. Qin H. Wang Z. Yu M. Liu Z. Peng H. (2021). Bone mesenchymal stem cell-derived sEV-encapsulated thermosensitive hydrogels accelerate osteogenesis and angiogenesis by release of exosomal miR-21. Front. Bioeng. Biotechnol. 9, 829136. 10.3389/fbioe.2021.829136 35127680 PMC8807520

[B34] Yoshida W. Matsugami D. Murakami T. Bizenjima T. Imamura K. Seshima F. (2019). Combined effects of systemic parathyroid hormone (1–34) and locally delivered neutral self‐assembling peptide hydrogel in the treatment of periodontal defects: An experimental *in vivo* investigation. J. Clin. Periodontol. 46 (10), 1030–1040. 10.1111/jcpe.13170 31292977

[B35] Yue K. Trujillo-de Santiago G. Alvarez M. M. Tamayol A. Annabi N. Khademhosseini A. (2015). Synthesis, properties, and biomedical applications of gelatin methacryloyl (GelMA) hydrogels. Biomaterials 73, 254–271. 10.1016/j.biomaterials.2015.08.045 26414409 PMC4610009

[B36] Zhang Y. Xu Y. Kong H. Zhang J. Chan H. F. Wang J. (2023). Microneedle system for tissue engineering and regenerative medicine. Exploration 3 (2023), 20210170. 10.1002/exp.20210170 37323624 PMC10190997

[B37] Zhang Y. Yu T. Peng L. Sun Q. Wei Y. Han B. (2020). Advancements in hydrogel-based drug sustained release systems for bone tissue engineering. Front. Pharmacol. 11, 622. 10.3389/fphar.2020.00622 32435200 PMC7218105

[B38] Zhao Y. Cui Z. Liu B. Xiang J. Qiu D. Tian Y. (2019). An injectable strong hydrogel for bone reconstruction. Adv. Healthc. Mat. 8 (17), 1900709. 10.1002/adhm.201900709 31353829

[B39] Zhao Y. Xing Y. Wang M. Huang Y. Xu H. Su Y. (2022). Supramolecular hydrogel based on an osteogenic growth peptide promotes bone defect repair. ACS omega 7 (13), 11395–11404. 10.1021/acsomega.2c00501 35415354 PMC8992256

