# Supplementary material for: Enhanced bone regeneration via local low-dose delivery of PTH1-34 in a composite hydrogel
Source: Front Bioeng Biotechnol. 2023 Jul 3;11:1209752. doi: 10.3389/fbioe.2023.1209752 (PMC10352085; doi:10.3389/fbioe.2023.1209752)
Supplement: Supplementary file 1 [file DataSheet1.pdf]

Supplementary Material

Enhanced Bone Regeneration via Local Low-dose Delivery of PTH_{1-34} in a Composite Hydrogel

Shanyong Zhang[1], Lei Ding[2], Gaoyang Chen[3], Jiayin Zhang[1], Wanbao Ge[1], Yuan Qu[1*]

[1]Department of Spine Surgery, The Second Hospital of Jilin University, Changchun, 130000, Jilin, China

[2]Department of Rehabilitation, The Second Hospital of Jilin University, Changchun, 130000, Jilin, China

[3]Shenzhen Key Laboratory of Musculoskeletal Tissue Reconstruction and Function Restoration, Department of hand Surgery, Shenzhen People's Hospital, The First Affiliated Hospital of Southern University of Science and Technology, Jinan University Second College of Medicine, Shenzhen, China

[*]Corresponding author: Dr. Yuan Qu, Department of Spine Surgery, The Second Hospital of Jilin University, Changchun 130000, Jilin, China, Email: quyuan0127@jlu.edu.cn

Table S1. The gelation time of different hydrogel systems.

**Hydrogel**	**GelMA：HepMA（V：V）**	**Gelation time (seconds)**
**1**	1：0	32 ± 1s
**2**	1：1	22 ± 2s
**3**	1：2	40 ± 4s
**4**	1：3	50 ± 5s
**5**	1：1 with Sr-HA	20 ± 2s

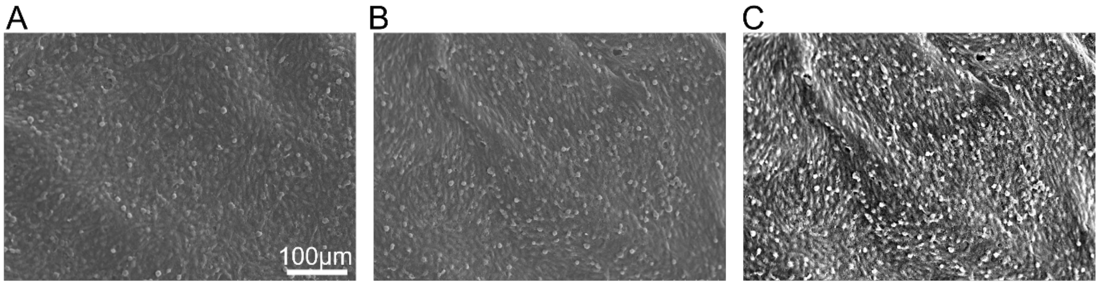

Figure S1. SEM characterization of MC3T3-E1 cell cultured on the surface of different hydrogels. (A:GelMA+HepMA hydrogel; B: GelMA+HepMA+SrHA hydrogel; C: GelMA+HepMA+SrHA/PTH_{1-34} hydrogel)

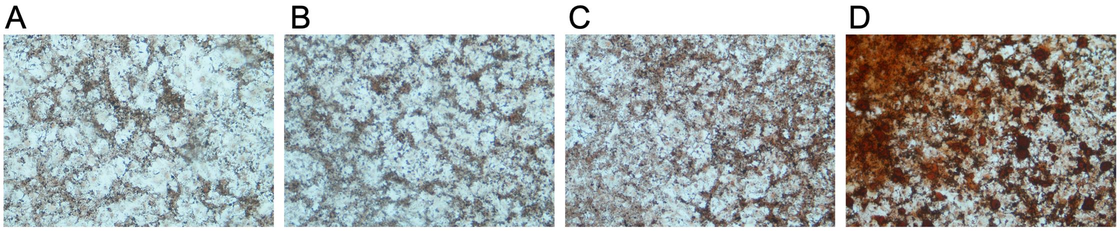

Figure S2. Alizarin red S staining of MC3T3-E1 cell cultured on the surface of different hydrogels. (A: Ctrl; B: GelMA+HepMA hydrogel; C: GelMA+HepMA+SrHA hydrogel; D: GelMA+HepMA+SrHA/PTH_{1-34} hydrogel)
